# A Comparative Study of Sleep Parameters in Opioid Dependent Patients on Opioid Substitution Therapy: Findings From India

**DOI:** 10.1192/bjo.2024.264

**Published:** 2024-08-01

**Authors:** Richa Tripathi, Ravindra Rao, Anju Dhawan

**Affiliations:** 1 All India Institute of Medical Sciences, Gorakhpur, India; 2 All India Institute of Medical Sciences, New Delhi, India

## Abstract

**Aims:**

Sleep problems are common in opioid users and in patients receiving opioid agonist treatment. The aim of the present study was to study the pattern and prevalence of subjective sleep disturbances in opioid dependent subjects maintained on opioid agonist treatment (buprenorphine and methadone).

Methods: A cross-sectional observational study was conducted in a tertiary health care center in India. 106 adult opioid dependent male patients maintained on buprenorphine and 50 adult opioid dependent male patients maintained on methadone who were initiated on medication at least six months prior, on stable dose of medication for last one month and were adherent on medication for at least 50% occasions in last one month were included in the study.

**Results:**

The mean age of the sample for buprenorphine-maintained group and methadone maintained group was 41.1 (SD: 14.3) years and 27.7 (SD: 7.8) years respectively. Tobacco, alcohol and cannabis were used by majority of the participants in both the groups. Most participants had used heroin by smoking before starting buprenorphine (n = 68, 64.1%) and methadone (n = 46, 88.5 %). The duration of use of illicit opioids was for median duration of 10 (IQR: 5, 22) years for buprenorphine group and 5 (IQR: 3, 7) years for methadone group.

In buprenorphine group, the participants had been on buprenorphine for a median duration of sixty (IQR: 17, 120) months. The mean current dose of buprenorphine was 10.2 (SD 3.8) milligram per day. The mean PSQI score was 6.6 (SD 3.4). About 63.2% (n = 67) of the participants have scores more than five (PSQI > 5) suggesting sleep problems. The mean subjective total sleep time of the sample was 403.5 (SD 94.8) minutes and median sleep latency was 35 (IQR 18.8, 62.5) minutes.

Similarly, in methadone group, the participants had been on methadone for a median duration of seventeen (IQR: 10, 22) months. The median current dose of methadone was 20 (IQR: 14, 36) milligrams per day. The mean PSQI score was 5.2 (SD 2.8). About 44.2% (n = 23) of the participants have scores more than five (PSQI > 5) suggesting sleep problems. The mean subjective total sleep time of the sample was 466.5 (SD 114) minutes and median sleep latency was 30 (IQR 15, 97.5) minutes. Subjective sleep problems were associated with past three months opioid use.

**Conclusion:**

The methadone group had relatively younger population with early onset of substance use. They were on relatively lesser dose of methadone. This group also had lesser sleep problems than the buprenorphine group.

